# Umbilical Cord Blood Transplants: Current Status and Evolving Therapies

**DOI:** 10.3389/fped.2020.570282

**Published:** 2020-10-02

**Authors:** Ashish O. Gupta, John E. Wagner

**Affiliations:** Division of Pediatric Blood and Marrow Transplant, Department of Pediatrics, University of Minnesota, Minneapolis, MN, United States

**Keywords:** umbical cord stem cells (UCSC), cord expansion, immune effector function, cord selection, alternative use of UCB

## Abstract

Hematopoietic cell transplants using stem cells from umbilical cord blood are used worldwide for the treatment of malignant and non-malignant disorders. Transplant procedures from this stem cell source have shown promising outcomes in successfully treating various hematologic, immunologic, malignant, and inherited metabolic disorders. Rapid availability of these stem cells is an important advantage over other unrelated donor transplants, especially in situations where waiting can adversely affect the prognosis. The umbilical cord blood is rich in CD34+ stem cells, though with a limited cell dose and usually takes longer to engraft. Limitations around this have been addressed by *in vivo* and *ex vivo* expansion techniques as well as enhanced engraftment kinetics. Development of adoptive immunotherapy using other components of umbilical cord blood such as regulatory T cells, virus-specific T cells, and natural killer cells has further transformed the field and enhanced the utility of umbilical cord blood unit.

## Introduction

Umbilical cord blood (UCB) stem cells have been successfully used for hematopoietic cell transplant (HCT) since the first report in 1989 (1). Over 40,000 UCB transplants have since been performed worldwide for a wide variety of malignant and non-malignant disorders (2, 3). The treatment outcomes of UCB transplant are comparable to those of related or unrelated bone marrow (BM) or peripheral blood (PB) used as graft source in hematologic malignancies (4, 5). In children with inherited metabolic disorders, outcomes are comparable for non-carrier-matched sibling BM and fully matched UCB (6, 7) and have been used in majority of transplants in this patient population (8). In other disorders such as primary immunodeficiency disorders, bone marrow failure syndromes, and hemoglobinopathies such as sickle cell disease and thalassemia, UCB transplant outcomes continue to improve (9–13). The establishment of cord blood banks have enabled safe storage and rapid availability of UCB stem cells for timely transplants for these disorders.

Cord blood banking was first established in 1993, and now about 5 million cord blood units are banked worldwide. About 800,000 of these are in public banks, and over 4 million UCB units are stored in private or family banks (3). The biologic properties of UCB units can be safely cryopreserved for more than 20 years under appropriate conditions, with efficient recovery of functional hematopoietic stem cell (HSC) and hematopoietic progenitor cells (HPCs) (14). The availability of these UCB banks has led to faster procurement of unrelated donor cord blood stem cells, significantly reducing the median search time from 3 to 4 months for bone marrow and peripheral blood stem cells to as early as 2 weeks for UCB stem cells (15). This is an important attribute which plays a role in choosing the appropriate donor HSC source for disorders where timing and flexibility are particularly critical such as high-risk malignancies and rapidly progressive inherited metabolic disorders. To maintain the optimum quality of these cord blood units, various organizations such as the National Marrow Donor Program and NetCord and Foundation for the Accreditation of the Cellular Therapy have established regulatory guidelines for the collection, processing, and storage of these units (16).

## Hematopoietic Properties

UCB is a rich source of primitive HSCs and progenitor cells. Though quantitatively limited, the proportion of highly proliferative HSCs is greater in UCB as compared to BM or PB allografts and has an enhanced capacity for homing and hematopoietic reconstitution (17, 18). These UCB cells also have higher capability for self-renewal, proliferation, and expansion under optimal conditions (19, 20). Higher expression of CD34+ antigen on their cell surface along with longer telomere length is an advantage. This provides a unique alternative to the invasive extraction of BM HSC and exposure to mobilizing agents prior to the collection of HSC in PB. As the collection of UCB takes nothing away from the neonate or the mother, safety is another major advantage.

### Immune Properties

With limited immunological memory in the neonate and high frequency of regulatory T cells that play an important role in maternal–fetal tolerance, these attributes likely play a critical role in the tolerability of human leukocyte antigen (HLA) mismatch in the recipients of UCB. However, studies also demonstrate that neonatal immune cells produce immune responses similar to that of adults in some aspects but not in others (21). Transplacentally acquired maternal antibodies play an important role in the initial neonatal defense against micro-organisms, followed by rapid maturation of T cells. Development of the maturation of neonatal T cells is facilitated by the cytokine milieu, with interleukin (IL)-2 mediating the differentiation of these naïve T cells into Tregs. These forkhead box p3 (Foxp3) + Tregs are present in higher proportion in neonatal lymph nodes as well as in umbilical cord blood (22, 23) and play an important role in immune tolerance in a developing fetus as well as in HLA-mismatched immune milieu.

### Impact of HLA Mismatch in Recipients of UCBT

The composition of cord blood from a relatively naïve immune system bestows the advantage of a lower frequency of alloreactive T cells in the graft. This results in a significantly lower incidence of graft vs. host disease (GVHD) in UCB recipients as compared to other graft sources (24). Because of these properties, less restrictive HLA matching criteria (HLA-A, HLA-B, and DRB1) are used for donor selection, thereby expanding the availability of UCB stem cells. However, previous studies have shown the impact of closely HLA-matched UCB units and cell dose on engraftment and risk of graft failure (25, 26). The role of total nucleated cell (TNC) dose and HLA matching was investigated by Barker et al. in 1,061 UCBT recipients which showed best transplant-related outcome and engraftment with 6/6 HLA-matched cord irrespective of TNC, while the effect of TNC was important in more mismatched cord recipients (27). The importance of enhanced matching, including HLA-C locus, was subsequently investigated by the combined Eurocord and Center for International Blood and Marrow Transplant Research (CIBMTR) study, analyzing the outcomes of 803 UCBT recipients in patients with hematologic malignancies (28). Significantly higher day 28 neutrophil recovery was noted in UCBT matched at HLA-A, HLA-B, HLA-C, and-DRB1 (8/8) compared to those mismatched at three or more HLA loci. More studies have since reported similar outcomes and the importance of allele-level HLA matching in UCBT recipients (29–31).

## Strategies to Enhance the Successful Use of UCB

UCB has been widely accepted as a useful HSC source; however, delayed hematopoietic recovery and consequent increases in initial hospital costs, concerns about delayed immunological recovery, and even the challenge of knowing how to select the best UCB unit for transplantation have been important barriers. This review will summarize the state of the art.

### UCB Unit Selection

The greater availability of high-quality and high-cell-content UCB units has resulted in increasingly improved engraftment and survival outcomes after UCBT. However, unit selection is often considered to be a major barrier to its use because multiple characteristics must be considered simultaneously. Several reports have previously outlined country-specific selection guidelines (32–37). This review provides a simplified step-by-step unit selection guide with additional principles for the selection of a cord blood unit (CBU) (Table 1). The guiding principle for CBU selection includes acceptable quality, adequate cell dose, and optimum high-resolution HLA matching. While not every transplant center experienced with UCB will use exactly the same selection criteria, the principles are uniform, with all centers recognizing the importance of finding the unit with a highly viable CD34 cell dose and HLA match at four of eight HLA antigens when possible, recognizing the importance of allele level typing at HLA-A, HLA-B, HLA-C, and DRB1.

**Table 1:** Umbilical cord blood unit search algorithm.

NMDP search algorithm	Step 1	Identify all CBUs that are HLA matched at 4/8 (considering A, B, C, and DRB1 at allele level based on haplogic) with a TNC dose >1.5 × 10^7^/kg filtering out those with a known CD34 dose <1.0 × 10^5^/kg)
	Step 2	List best to worst HLA match
	Step 3	Within each HLA match category list highest to lowest by NC dose
	Step 4	Provide information on CD34 dose, ABO types, race/ethnicity and any missing identity or history information
Center specific filters	Step 5	Center's will be able to adjust filters and how units are sorted: (a) Restrict or relax HLA match (e.g., permit 3/8 or eliminate <5/8) (b) Relax CBU age (e.g., include older units >10 years) (c) Restrict or relax eligible CBBs (e.g., include those that are not FACT accredited, or located out of country) (d) Relax RBC replete status (e) Eliminate HLA antigens (based on recipient anti-HLA antibodies) (f) Change sort and simply list units based on highest to lowest cell dose and not group by HLA match
Additional principals		(1) RBC replete units are not recommended as these have been associated with more adverse events including life-threatening infusion reactions (2) Consider cryovolume for units expected to undergo post-thaw dilution (3) Focus on units with attached segments for confirmatory typing (if not available, consider rapid HLA screen if possible at time of unit thaw and prior to infusion) (4) Perform minimum of 8 high-resolution (HLA-A, HLA-B, HLA-C, and HLA-DRB1) for both patient and CB unit (5) While balancing CD34 cell dose and HLA matching, the greater the HLA mismatch, the higher cell dose is needed for a successful outcome

*CBU, cord blood unit; CBT, Cord blood transplant; HLA, human leukocyte antigen; TNC, total nucleated cells; CBBs, cord blood banks; RBC, red blood cell*.

## Strategies for Early Hematopoietic Recovery

### *In vivo* Expansion

The use of double UCB resulting in *in vivo* expansion of stem cells and thereby improving engraftment was first shown by Barker et al. (38). Since then, other strategies for *in vivo* UCB expansion have been used, such as haplo-cord transplants where a small dose of haploidentical stem cells is used for early engraftment and UCB expansion in the optimal cytokine milieu (47). However, with these *in vivo* expansion techniques, there is a concern of higher risk of graft-versus-host disease (GvHD) and prolonged mixed chimerism, though studies have shown otherwise (48, 49).

#### Double Cord Blood Transplants

The use of two umbilical cord blood units *in vivo* has shown promising outcomes and has helped obviate some of the limitations of single UCB HCTs in older children and adults. The two UCB units can be either infused unmanipulated or one unit can be infused unmanipulated and the other one selected for HSCs and HPCs. In majority of patients, one unit predominates either by rejection by the other unit or having a competitive advantage with higher CD34+ stem cells (50). Overall HCT outcomes with double UCBT (dUCT) have been comparable to matched-related and unrelated outcomes (51).

#### Haplo-Cord Transplants

This approach combines infusion of a smaller dose, but well-matched, of UCB unit together with mobilized PB CD34+ cells from a haploidentical donor. In this setting, early engraftment and hematopoietic recovery is achieved by usually the haploidentical donor, which then subsequently yields sustained and durable engraftment to the UCB unit. Outcomes of this approach using both myeloablative and reduced-intensity conditioning regimens have shown reduced infectious and immunologic complications with good outcomes (49, 52). This approach can be useful for adults with limited matched unrelated donor and UCB availability where a haploidentical donor is available, allowing the use of a single UCB.

### UCB HSPC Expansion

Various approaches have been used to expand HSCs and HPCs from UCB with the use of feeder stromal cells, epigenetic modifiers, and small molecules. Over the past decade, UCB expansion has been successfully achieved, demonstrating rapid recoveries of neutrophils and platelets, better rates of engraftment, and fewer days in the hospital as surrogate for reducing costs.

#### *Ex vivo* Expansion Culture

Several *ex vivo* expansion strategies used over the last few years show promising results today (Table 2). Irrespective of the technique, there is a robust increase in CD34+ stem cells and their progenitors, leading to much faster neutrophil recovery and myeloid engraftment after infusion as compared to historical controls (39–44). A regimen containing different cytokines or small molecules is used to exponentially enhance the stem cell population, thereby influencing the kinetics of engraftment and reducing the duration of severe neutropenia.

**Table 2:** Clinical trials for *ex-vivo* cord blood stem cell expansion.

**Study**	**Expansion technique**	**Model**	**Number of Patients**	**Duration of culture**	**Median CD34 cell dose after expansion (× 10^**6**^)/kg**	**Median CD34 expansion (folds)**	**Neutrophil engraftment (in days, range)**	**Platelet engraftment (in days, range)**
Delaney et al. (39)	Notch ligand delta 1	Double UCBT	10	16 days	6.1 (0.9–13.6)^*^	164 (41–471)	16 (7–34)	NA
de Lima et al. (40)	Copper chelation (TEPA)	Double UCBT	8	21 days	9.4 (0.39–247.7)	161 (2–620)	30 (16–46)	48 (35–105)
Stiff et al. (41)	Copper chelation (TEPA)	Double UCBT	101	21 days	1.028 (0.137–939.6)	77	21 (18.4–23.5)	54 (43.3–61.9)
Horwitz et al. (42)	Nicotinamide	Double UCBT	11	21 days	3.5 (0.9-18.3)	72 (16–86)	13 (7–26)	33 (26–49)
Horwitz et al. (43)	Nicotinamide	Single UCBT	36	21 ± 2 days	6.3 (1.4–14.9)	33	11.5 (9–14)	34 (32–42)
Wagner et al. (44)	StemRegenin 1	Double UCBT	17	21 days	17.5 (1.4–48.3)	248 (66–446)	15 (6–30)	49 (28–136)
de Lima et al. (45)	MSC co-culture	Double UCBT	31	7 days	0.95 (1.6–9.34)	30	15 (9–42)	42 (15–62)
Cohen et al. (46)	UM 171	Double UCBT/ Single UCBT	27 (4 received double UCBT)	7 days	2.87 (1.91–3.96)	28 (19–35)	18 (12.5–20)	42 (35–47)

* *Reported mean*.

*TEPA, tetraethylenepentamine; UCBT, Umbilical cord blood transplant; MSC, mesenchymal stem cell*.

In early clinical trials with *ex vivo* expanded UCB, investigators often first evaluated the engraftment potential of the expansion product in the context of double UCBT, where one unit was expanded and one was infused without prior manipulation. The notch signaling pathway has an important regulatory role in hematopoietic differentiation as well as in the proliferation of HSCs and HPCs (53). Using engineered notch ligand delta 1, the expanded UCB unit resulted in rapid neutrophil recovery, but long-term engraftment was most often from the unmanipulated UCB unit (39). This approach demonstrated safety and feasibility as well as early neutrophil recovery [16 days; range (r) 7–34 days] as compared to the median time of 26 days (r, 16–48 days; *p* = 0.002) in historical controls with double UCBT without expansion (38).

Co-culture with mesenchymal stem cells (MSCs) to provide the necessary factors for HSC expansion was explored as an *ex vivo* expansion strategy (45). It used an initial co-culture with MSCs for 7 days, followed by culture with cytokines. This trial enrolled 31 adults who underwent dUCBT, one with expanded cord and another with an unmanipulated cord. About 30-fold higher CD34+ cell dose was noted in the expanded unit. This study also reported earlier neutrophil and platelet recovery as compared to the CIBMTR data for dUCBT. The expanded cord lasted for about a year, when entire donor chimerism was noted from the unmanipulated cord. This technique has been further refined by the addition of fucosylation during UCB expansion (clinical trial NCT03096782) to investigate further improvement in hematopoietic recovery.

While promising, the results of these two studies suggested that these culture methods preferentially expanded primitive progenitors at the expense of HSC, providing only a transient wave of hematopoietic recovery. However, in the absence of T cells after expansion culture, the expanded product is at an immunological disadvantage in the setting of dUCBT, where one unit actively rejects the other (54). Based on this observation, transient engraftment after expansion culture may have been due to the absence of T cells rather than the loss of HSC. Therefore, subsequent expansion trials re-cryopreserved the CD34-depleted fraction after the CD34-enriched population was placed in expansion cultures.

Copper chelation technique using tetraethylenepentamine (TEPA) was investigated, with pre-clinical evidence demonstrating the prevention of stem cell differentiation in an *in vitro* culture (55). The UCB graft is derived from a single unit, where a fraction of the UCB unit undergoes a 21-days expansion culture in the presence of TEPA, followed by infusion of expanded and unmanipulated fractions on transplant day. The phase I/II clinical trial enrolling 10 patients confirmed safety and feasibility and showed neutrophil engraftment at day 30 (r, 16–46) and platelet engraftment at day 48 (r, 35–105 days) (40). A larger international multi-center trial was then conducted and reported results from 101 patients, which showed a median nucleated cell expansion of about 400-fold, with CD34 expansion of 77-fold (41). The 100-days survival was superior to dUCBT in the contemporary period. Neutrophil and platelet engraftments were significantly earlier than the comparison group (21 vs. 28 days and 54 vs. 105 days, respectively).

More recent trials evaluated small molecules, including vitamin B derivatives, aryl hydrocarbon receptor antagonists (AHRa), and pyrimidoindole derivatives that impeded HSC differentiation in cultures containing stimulatory cytokines, like SCF, Flt-3L, and thrombopoietin, but also infused the unit's T cells, in contrast to prior trials. Nicotinamide, a vitamin B derivative, inhibits differentiation, thereby enhancing the expansion of HSC and HPCs expanded in *ex vivo* cultures with stimulatory hematopoietic cytokines. In the initial phase I trial, 11 patients were enrolled. Of the 11 patients, the median time to neutrophil and platelet recovery was 13 and 33 days, respectively, faster than the controls (25 days, *p* < 0.001 and 37 days, *p* = 0.085). However, it is most notable that sustained myeloid engraftment from the NiCord-derived unit was observed in eight patients (42). Subsequently, a phase II study was completed using NiCord as a stand-alone graft in 36 patients (median age, 44 years), with high-risk hematologic malignancies treated with myeloablative conditioning. The results were compared to those of 146 patients who received standard UCB transplantation, with data reported to the CIBMTR. In the recipients of NiCord, the cumulative incidence of neutrophil engraftment was 94% at day 42, and the median time to neutrophil recovery was 11.5 days (95% CI, 9–14) vs. 21 days (95% CI, 20–23) for patients who received standard transplant (*p* < 0.001). Similarly, the median time to platelet recovery was 34 days (95% CI, 32–42) with NiCord vs. 46 days (95% CI, 42–50) with standard UCB (*P* < 0.001). The unadjusted probability of overall survival after 2 years was 51% (95% CI, 33–67), and the 2-years disease-free survival was 43% (95% CI, 24–60) (43).

Boitano et al. reported the first use of an AHRa, StemRegenin 1 (SR1), for purified CD34+ expansion when cultured in media with SCF, Flt3L, TPO, and IL6, resulting in about a thousand-fold expansion (56). In the initial phase I/II clinical trial, 18 patients were treated with the lower dose unit placed in expansion culture and the larger dose unit which was unmanipulated. All the patients demonstrated sustained engraftment. In the 12 that had unit predominance with the expanded unit, the median time to recovery was 10.5 days, in contrast to 23 days in those engrafted with the unmanipulated unit (44). With sustained engraftment in the 12 patients recovering with the expanded product, the subsequent study evaluated the safety and the efficacy of the expanded product as a stand-alone graft. In addition, because of the marked expansion with the AHRa, lower-dose UCB units containing a cell dose of 1 × 10^7^ nucleated cells, rather than 3 × 10^7^ nucleated cells, per kilogram of recipient body weight were considered, potentially increasing the chance of better HLA-matched units for adults. An interim analysis demonstrated CD34+ cell expansion of 421-fold (r, 219–1,476), with the patients receiving a median of CD34+ cell dose of 2.6 × 10^7^/kg (r, 0.9–13.5 × 10^7^/kg) and CD34+CD90+ cell dose of 1.3 × 10^6^/kg (r, 0.5–7.0 × 10^6^/kg). Neutrophil recovery occurred in 100% of patients at a median of 13 days (*r*, 8–31) vs. 25 days in prior recipients of unmodified CB (*p* < 0.01). Similarly, platelet recovery and red blood cell transfusion independence occurred in 100% at a median of 36 days (r, 30–56) and 48 days (r, 13–196), respectively. Time to neutrophil and platelet recovery strongly correlated with CD34+CD90+ dose, and there had been no transplant-related mortality reported so far (57).

The pyrimidoindole derivative, UM171, was evaluated as another strategy for UCB HSC expansion which enhances the self-renewal potential of human long-term repopulating HSCs independently of AHR suppression (58). The clinical phases 1 and 2 trial using this compound was conducted in two parts (46). Part 1 enrolled four patients who received dUCBT—one with unmanipulated UCB unit and the other expanded with UM171—until the patients showed UM171 UCB unit-derived engraftment. In part 2 of this study, 22 patients received single UM171 expanded UCBT with a dose de-escalation design. The minimal UCB unit that achieved prompt engraftment as a single UM171-expanded UCBT was 0.52 × 10^5^ CD34+ cells. The median time to neutrophil (ANC > 500/μl) and platelet recovery was 18 (r, 12.5–20 days) and 42 days (interquartile range, 35–47), respectively, with no incidence of graft failure.

#### Augmenting Homing

Based on preclinical models, it is clear that relatively few HSCs make it to the hematopoietic niche. Therefore, investigators explored ways that might augment homing and engraftment as an alternative to expansion culture. The first studies evaluated direct intra-bone marrow injection (IBMI) of UCB stem cells (59). This phase I/II study enrolled 32 adult patients with acute leukemia. The median time to neutrophil and platelet recovery was 23 and 36 days, respectively, and early sustained donor-derived engraftment was noted among all patients. There was no incidence of grade III and IV acute GVHD on this study. In a subsequent study by Brunstein et al., a dUCBT platform was used for the IBMI of one of the UCB units, while the other was given intravenously (60). Ten adult patients were enrolled on this trial, and the median time to neutrophil and platelet recovery was 21 and 69 days, respectively. In nine out 10 patients that engrafted, four engrafted with IBMI UCB unit. The trial demonstrated the safety of the procedure, but the technique offered no advantage over the traditional intravenous route.

Alternatively, agents like the dimethylated form of prostaglandin E2 (dmPGE2) and fucosylation have been used to augment the homing of HSCs (61). In the earlier approach, dmPGE2 was used to augment the homing of stem cells by increasing the number of stem cells that reach the bone marrow niche. This was considered to deliver a greater number of stem cells to the target site without the need for *in vivo* or *ex vivo* expansion (61). A phase I safety and efficacy trial was conducted, evaluating this concept using co-transplantation of a dmPGE2-treated UCB with an unmanipulated cord in patients with hematologic malignancies (62). The trial initially enrolled nine patients, with median time to neutrophil and platelet engraftment at 24 and 72.5 days, respectively. Two of seven patients undergoing engraftment demonstrated prolonged hematopoiesis from the dmPGE_2_-UCB units. Given the lack of accelerated engraftment in the initial trial, dmPGE2 was optimized in the subsequent trial with a modulation protocol, and 12 additional patients were enrolled. The median time to neutrophil engraftment was 17.5 days (r, 14–31 days) compared to 21 days for the historical cohort (*p* = 0.045). The median time to platelet engraftment was 43 days (r, 26–60 days), and 10 of 12 patients had early and sustained engraftment of the dmPGE_2_-UCB unit.

Another approach was evaluated by exploring the role of complement 3a (C3a), which attaches to the HSCs and improves homing by its immunomodulatory properties, including stromal-derived factor I (SDF I)-mediated homing (63). Based on pre-clinical data, the phase I study was conducted in adults receiving non-myeloablative conditioning in a dUCT model. Engraftment was noted in two-thirds of the patients from the non-manipulated cord, thus failing to show earlier homing and engraftment with this technique (64). Another group investigated the effect on homing with inhibition of dipeptidyl peptidase (DPP)-4, which is a peptide cleavage protein that truncates the chemotaxis factor, SDF-1-alpha. A pre-clinical investigation in mice demonstrated that deletion or inhibition of DPP-4 enhanced engraftment of human CD34+UCB cells in mouse marrow (65, 66). In the subsequent clinical trial, an oral inhibitor of DDP-4, sitagliptin, was used to enhance the engraftment of single-unit UCB transplants in adults with high-risk hematological malignancies (67). In this feasibility trial, 24 patients received sitagliptin on days 1 and 2 at a dose of 600 mg daily and engrafted at a median of 21 days (r, 13–50). Though sitagliptin was well-tolerated, a significant reduction in area under the curve was noted. After dose optimization, 600 mg every 12 h administered on days −1 to +2, another 15 adult patients were treated, and all engrafted by day 30, with 12 (80%) engrafting by day 21 (68). The median time to neutrophil engraftment was 19 days (r, 12–30).

Slower homing and engraftment with UCB relative to bone marrow HSC had also been attributed to poor binding to adhesion molecules P- and E-selectins present on bone marrow endothelial cells (69). Pre-clinical models showed that both endogenous as well as *ex vivo* fucosylation of UCB HSCs increased the affinity for these adhesion molecules, resulting in earlier engraftment (70, 71). In the phase-I clinical trial using the dUCT model, one unit underwent fucosylation using fucosyltransferase-I enzyme, while the other unit was infused unmanipulated. Significantly faster neutrophil (17 vs. 26 days; *p* < 0.05) and platelet engraftment (36 vs. 46 days; *p* < 0.05) was noted from both units compared to the historical controls, suggesting that endogenous fucosylation benefited the engraftment of the unmanipulated cord as well (72).

### Alternative Uses of UCB

#### Regulatory T Cells for Prevention of Immune Reactivity

GVHD has been one of the most serious complications for patients undergoing allogeneic HCT. The complex interaction between donor immune cells and residual host immunity results in extensive tissue damage requiring prolonged immune suppression, thereby increasing the risk of infection. Different strategies targeting *in vivo* or *ex vivo* T cell depletion have been shown to reduce the risk of GVHD, but these T cells also assist with engraftment and hasten immunologic recovery.

UCB has unique immunologic properties as it unites the maternofetal hematologic and immune environment and is one of the best examples of immune tolerance. This led to the identification of immunomodulatory cells in the UCB which dampen the pro-inflammatory immune response of the activated T cells. These T cells, also known as regulatory T cells (Tregs), are CD4+CD25+Foxp3+ and can proliferate in the presence of IL-2 (73). These Tregs have since shown to play an important role in autoimmune diseases (74, 75) as well as in regulating systemic pro-inflammatory response such as GVHD (76, 77). Edinger et al. demonstrated the role of CD4+CD25+Tregs in inhibiting the GVHD while preserving the graft-versus-tumor effect in mice with leukemia and lymphoma (78). Pre-clinical studies investigating the UCB components demonstrated the role of Tregs within the UCB, responsible for maternal–fetal tolerance (79, 80). These UCB-derived Tregs can be successfully isolated and expanded *ex vivo* to about 100-fold using anti-CD3/28 monoclonal antibody (mAb) along with supplemental IL-2 (81).

Early clinical trials investigating the safety profile of UCB-derived Treg infusion in adults with malignant disorders showed an encouraging safety profile and reduced grades II–IV acute GVHD rates (43 vs. 61%; *p* = 0.05) when compared to the historical cohort (82). Much greater *in vitro* expansion of isolated Tregs was obtained using the artificial antigen-presenting cells (aAPCs) to about 1,250-fold in 2.5–3 weeks compared to anti-CD3/CD28 mAb beads (83). These expanded UCB Tregs using modified aAPCs expressing OX40 and 4-1BBL had significantly better survival without loss of suppressor potency. A second dose expansion trial with Tregs showed a significant reduction of grades II–IV acute GVHD (45 vs. 9%; *p* = 0.05) compared to the historical controls, with no dose-related infusional toxicity or adverse reactions (84).

#### Virus-Specific T Cells

Viral infections after HCT result in substantial morbidity and mortality. Cytomegalovirus, Epstein–Barr virus, and adenovirus constitute the majority of significant viremias after HCT. Transplant-related variables that contribute to the risk include the underlying disease, donor and graft source, preparative regimen, and the degree of T cell depletion (*in vivo* or *ex vivo*) (85, 86). Current pharmacologic antiviral prophylaxis and treatment are limited in efficacy and are toxic to various organs (87, 88). The use of adoptive virus-specific T cell therapy was initially reported in 1992 by Ridell et al. (89) and has since developed and is increasingly used in HCT patients at high risk of organ toxicities or who have failed in conventional therapies. Readily available “off-the-shelf” third party is now available, targeting multiple viral infections (90–94), which is the major advantage these UCB-derived virus-specific T cells offer over other sources. However, the inability to do T cell expansion after UCBT and the naivety of UCB-derived T cells with limited priming resulting in a blunted immune response (95, 96) continue to be the main challenges.

#### UCB-Derived NK Cells

Natural killer (NK) cells are innate lymphocytes characterized by CD16+/CD56+ surface proteins and play an important role in identifying non-self-antigens without preemptive exposure. These are not antigen specific and are capable of identifying cells with reduced major histocompatibility complex expression as well. Their response is regulated through a balance between activating and inhibitory signals derived from surface receptors on the cell membrane that engage with related ligands on target cells (97, 98). The inhibitory killer cell immunoglobulin-like and NKG2A family of receptors recognize self HLA class I antigens, while the activating receptors include the natural cytotoxicity receptors NKp46, NKp30, NKp44, CD16, NKG2D, NKG2C, DNAX accessory molecule-1, and 2B4 recognize viral proteins as well as antibodies on target cells (99–101). NK cells also express checkpoint inhibitory receptors which further play an important role in antitumor activity and prevent disease relapse after HCT. In addition to their cytotoxic effect on target cells, their interaction with other immune cells, like dendritic cells and T cells, further potentiates the overall immune response.

NK cells are the first ones to reconstitute after HCT, providing an important immunologic barrier to invading pathogens in this critical period and especially more so after UCBT compared to other donor sources (102). UCB-derived NK cells are noted to express higher inhibitory receptors and fewer activating receptors in the early post-transplant period. Though these NK cells have high proliferative capacity, they are immature in function and have relatively less cytotoxic potential (103–105). Despite these issues, NK cells continue to play an important role in early post-HCT period, and their role in facilitating engraftment has been demonstrated in murine models (106, 107). This supports the premise of using *ex vivo*-expanded NK cells after UCB infusion to enable early engraftment and reduce the duration of neutropenia. Another important feature of NK cells in the post-HCT period is their strong potential for antitumor activity. The role of NK cell alloreactivity and kir-mismatch in antitumor activity and preventing acute myeloid leukemia relapse was first demonstrated by Ruggeri et al. (108). The interaction of NK cells with other immune cells and the direct cytotoxic effect on tumor cells play an important role in reducing tumor relapse, which has since been shown by other studies (109, 110). This again supports the role of early NK recovery and infusion of expanded NK cells after UCBT.

A major challenge with UCB-derived NK cells is the limited number of available cells in a cord blood unit. Isolation and *ex vivo* expansion using an optimal cytokine cocktail (IL-2- or IL-7-mediated) thus become crucial to increase the cell dose (111–113). The expansion techniques under good manufacturing practices are now well-established, providing an important tool for adoptive immunotherapy against hematologic malignancies (114, 115) as well as solid tumors (116, 117). NK-cell-based immunotherapy continues to be a developing area with ongoing research on enhancing NK cell function by the increased expression of activating receptors or blocking inhibitory receptors (118), overcoming tumor resistance by blocking inhibitory signals and better understanding of the NK cell–tumor interactions to develop targeted immunomodulation.

In the recent phase I/II study by Liu et al., 11 patients with CD19+ hematologic malignancies (relapsed or refractory) were administered HLA-mismatched anti-CD19 CAR-NK cells derived from UCB (119). These HLA-mismatched NK cells were transduced using a retroviral vector expressing genes that encode anti-CD19 CAR, IL-15, and inducible caspase 9 as a safety switch, expanded *ex vivo*, and infused at different doses after lymphodepleting chemotherapy. There was no increase in the levels of inflammatory cytokines post-infusion and no incidence of cytokine release syndrome, neurotoxicity, or GVHD. Eight patients tolerated the infusion, with seven demonstrating complete and rapid response within 30 days of infusion. The infused CAR-NK cells expanded and persisted at low levels for at least 12 months, which was possibly supported by IL-15 in the construct as well as the lymphodepleting chemotherapy. These preliminary results from this study paves the way forward for the development of off-the-shelf NK-CAR products using UCB.

### Other UCB-Derived Products

Induced pluripotent stem cells (iPSCs) can be derived from the UCB unit and now have been investigated in the development of multiple regenerative therapies including the skin, cartilage, neurodegenerative and spinal cord injuries, ocular degenerative diseases, and musculoskeletal disorders among many (120, 121). As regenerative therapies continue to develop, these UCB-derived iPSCs will continue to play a major role with their unique properties and easy availability. The role of UCB in immune modulation and regeneration is also being explored in several non-hematologic and non-malignant diseases. Currently, autologous or allogeneic UCB units are being investigated under ongoing clinical trials for hypoplastic left heart (NCT01856049 and NCT01883076), acute ischemic stroke (NCT03004976), cerebral palsy (NCT01072370), and hypoxic neurologic injuries (NCT03526588).

### Future Directions

**Figure 1:**
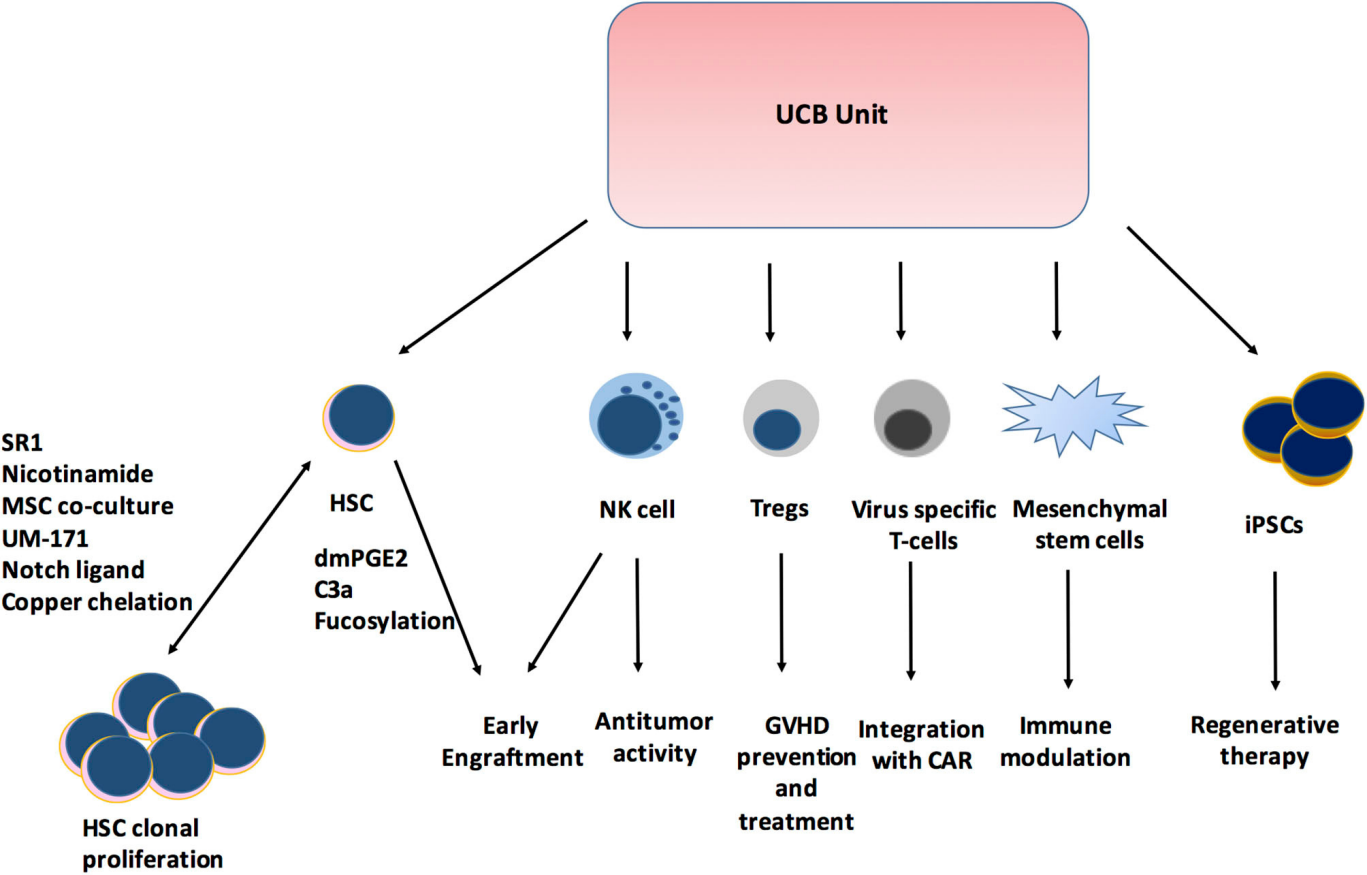
Expanding role of Cord blood unit. SR1, StemReginin1; HSC, hematopoietic stem cell; dmPGE2, dimethyl prostaglandin E2; C3a, Complement 3a; NK cell, Natural killer cell; Tregs, Regulatory T cells; GVHD, Graft versus host disease; CAR, Chimeric antigen receptor; iPSCs, induced Pleuripotent stem cells.

The use of UCBT has evolved from being limited to HCT for pediatric patients to expanding the role in adoptive cellular therapy (Figure 1). Remarkable research in the field has helped overcome the limitations of UCB with stem cell expansion and significantly improved hematopoietic and immunologic recovery, which has led to a shorter duration of hospitalization and hence lesser healthcare resource utilization. The outcomes of UCBT continue to improve with these newer modalities. In this new era of UCB-derived cells, the role of *in vivo* and *ex vivo* UCB expansion, tolerant Tregs, expansion of multi-virus-specific T cells, NK cells, and iPSCs will be crucial. The expanding use of UCB-derived immunotherapy will play an important role beyond HCT and continue to improve disease outcomes.

## Author Contributions

AG performed literature review and drafting of manuscript. JW provided critical review of manuscript, contributed further literature, and finalized the manuscript. All authors contributed significantly for preparation of this manuscript.

## Conflict of Interest

The authors declare that the research was conducted in the absence of any commercial or financial relationships that could be construed as a potential conflict of interest.
